# Retraction: MiR-744 increases tumorigenicity of pancreatic cancer by activating Wnt/β-catenin pathway

**DOI:** 10.18632/oncotarget.28806

**Published:** 2025-12-31

**Authors:** Wei Zhou, Yongfeng Li, Shanmiao Gou, Jiongxin Xiong, Heshui Wu, Chunyou Wang, Haijiao Yan, Tao Liu

**Affiliations:** ^1^Pancreatic Disease Institute, Union Hospital, Tongji Medical College, Huazhong University of Science and Technology, Wuhan, Hubei, 430022, P.R. China; ^2^Department of Oncology, the Third Affiliated Hospital of Soochow University, Changzhou, 213003, P.R. China; ^3^Department of Digestive Surgical Oncology, Union Hospital, Tongji Medical College, Huazhong University of Science and Technology, Wuhan, Hubei, 430022, P.R. China

**This article has been retracted:** Oncotarget has completed its investigation of this paper. Image forensic analysis confirmed that one panel in Figure 2C and three panels in Figure 3C were duplicated from corresponding panels in Figures 2C and 3C of reference [1], published earlier, despite representing different experiments. In addition, the miR-744 sequence shown in Figure 5A is incorrect and does not match the sequence listed in miRBase. The authors initially provided data for potential corrections; however, the extent and number of required corrections exceeded a reasonable threshold. As a result, the Editorial Decision was made to retract the publication. The National Natural Science Foundation of China Supervision Committee also confirmed misconduct, determining that the first author (Zhou Wei), second author (Li Yongfeng), and corresponding author (Liu Tao) were responsible for the identified issues. The authors agreed with the retraction.

Original article: Oncotarget. 2015; 6:37557–37569. 37557-37569. https://doi.org/10.18632/oncotarget.5317
